# Sexual reproduction and genetic exchange in parasitic protists

**DOI:** 10.1017/S0031182014001693

**Published:** 2015-02

**Authors:** GARETH D. WEEDALL, NEIL HALL

**Affiliations:** Institute of Integrative Biology, Biosciences Building, Crown Street, University of Liverpool, Liverpool L69 7ZB, UK

**Keywords:** parasites, protists, sex, meiosis

## Abstract

A key part of the life cycle of an organism is reproduction. For a number of important protist parasites that cause human and animal disease, their sexuality has been a topic of debate for many years. Traditionally, protists were considered to be primitive relatives of the ‘higher’ eukaryotes, which may have diverged prior to the evolution of sex and to reproduce by binary fission. More recent views of eukaryotic evolution suggest that sex, and meiosis, evolved early, possibly in the common ancestor of all eukaryotes. However, detecting sex in these parasites is not straightforward. Recent advances, particularly in genome sequencing technology, have allowed new insights into parasite reproduction. Here, we review the evidence on reproduction in parasitic protists. We discuss protist reproduction in the light of parasitic life cycles and routes of transmission among hosts.

## INTRODUCTION

Several species of single-celled eukaryotes are important causes of death and disease in humans and domestic animals. These include such ancient scourges as the malaria parasites; trypanosomes, agents of sleeping sickness and Chagas disease; *Leishmania* parasites and numerous agents of primarily intestinal disease of humans and animals, including *Cryptosporidium, Entamoeba* and *Giardia*. Understanding the biology, and particularly the life cycles, of these parasites has been an important step in combating the diseases they cause. However, despite considerable research, many aspects of their biology remain mysterious.

A key part of the life cycle of an organism is reproduction. In protists, particularly parasitic protists, the sexuality or otherwise of numerous species has been hotly debated for many years (Tibayrenc *et al.* 1990; Tibayrenc and Ayala, 2002). More recently, particularly with the advent of high throughput DNA sequencing technologies, evolutionary and population genomics have provided new evidence about reproduction in these organisms.

Here, we review some recent studies of sex and genetic exchange in parasitic protists and consider how the various reproductive modes of unicellular parasites may have adapted to differing parasitic lifestyles. Parasites considered in this review include important representatives of several major clades. Apicomplexans include malaria parasites of the genus *Plasmodium* as well as *Babesia* and *Theileria* and intestinal parasites of the genera *Cryptosporidium, Toxoplasma* and *Eimeria*. The Excavata include kinetoplastid parasites of the genera *Trypanosoma* and *Leishmania* as well as diplomonad parasites of the genus *Giardia* and parabasalid parasites of the genus *Trichomonas*. The Amoebozoa include enteric parasites of the genus *Entamoeba*.

## SEX IN PARASITIC PROTISTS

True sex, consisting of cell fusion, nuclear fusion and meiosis is found only in eukaryotes. It is closely linked to the exchange and recombination of genetic material among individuals because it brings DNA molecules with different genealogies into contact, so that crossing over and exchange may occur. It has been argued that the evolution of sex is intimately associated with the origin of eukaryotes and arose much later than recombination, which occurs in all organisms (Cavalier-Smith, 2002, 2010).

Sex is widespread among eukaryotes and the reason for its maintenance is a central question of evolutionary biology. The benefits of sex, such as purging the genome of deleterious mutations and bringing together advantageous mutations, must be set against its fitness costs: mates have to be found, special cell types formed and diploid genomes maintained (Lehtonen *et al.* 2012). Indeed, the cost-benefit ratio may differ radically among species where, for instance, massive population sizes in micro-organisms (and the associated increase in the strength of selection) might obviate the need for sex to avoid Muller's ratchet, the irreversible accumulation of deleterious mutations. Some eukaryotes appear to have lost the ability to reproduce sexually altogether, such as some species of *Daphnia* and aphids (Innes and Hebert, 1988; Delmotte *et al*. 2001) but ancient asexuality appears to be rare (see Schurko and Logsdon, 2008).

The term ‘protists’, used to encompass numerous groups of species, can be misleading unless we remember just how vast the evolutionary distances between them can be (Baldauf, 2003). The protists are not monophyletic and genetic distances between the different protist phyla are many orders of magnitude greater than that between fungi and mammals, for example. Of the parasitic protists considered here, the Apicomplexa include the genera *Plasmodium, Babesia, Theileria, Toxoplasma, Eimeria*, and *Cryptosporidium*; the Euglenozoa include *Trypanosoma* and *Leishmania*; the Fornicata include *Giardia*; the Parabasalia include *Trichomonas*; and the Amoebozoa include *Entamoeba*.

In discussing sex in the parasitic protists, two features should be considered: firstly, their status as protists; and secondly, their status as parasites.

The common feature of protists is their unicellularity, which makes them fundamentally different to multicellular organisms. In terms of their reproduction, both mitotic and meiotic cell divisions are reproduction, in that they both produce new individual cells. It is, therefore, entirely possible that reproduction is achieved only by mitosis. Another ‘unusual’ aspect of protists is that they do not conform to life-patterns familiar from multicellular organisms: diploid cells dividing by mitosis, interspersed with haploid gametes. Even obligate sexual protists, such as *Plasmodium*, spend the majority of their life cycle as haploid cells with only a brief spell of diploidy prior to meiosis. Therefore, meiosis may not be required for reproduction in some species that are able to complete their life cycles by clonal reproduction. In other species the haploid forms may be integral to the completion of the life cycle.

**Fig. 1.**
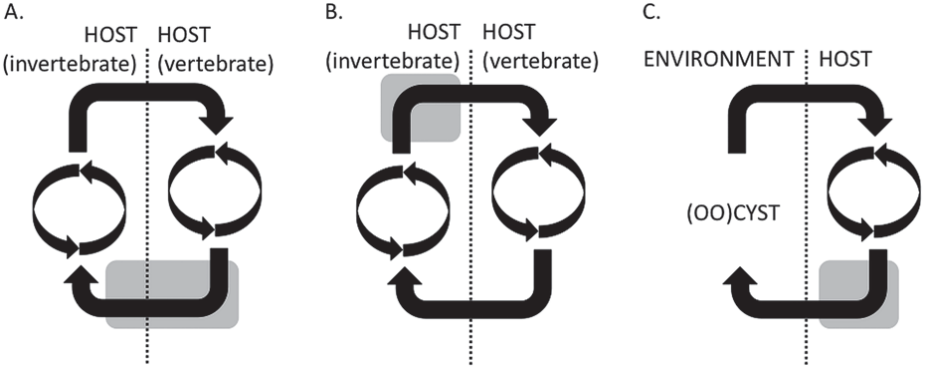
Simplified schematic representations of the life cycles of vector- and fecal–oral-transmitted parasites. (A) The *Plasmodium falciparum* life cycle, which has two cycles of asexual growth, one in each host, and the sexual stages (grey shading) which span transmission from one host to another; (B) the *T. brucei* life cycle, in which the sexual stages appear to take place in the insect host, after a cycle of asexual cell divisions but before transmission to the mammalian host; (C) the *Cryptosporidium* and *Eimeria* life cycle, in which the sexual stages all take place within the single host and cysts are passed into the environment.

The second consideration is parasitism, a derived state that has arisen independently in many species. The evolutionary consequences of this are that many adaptations to the particular selective pressures of parasitism may have similar, independently derived results in diverse species. So distantly related parasites in similar niches may show similar adaptations while closely related lineages have evolved very different life cycles. This is shown in Fig. 1, which illustrates the life cycles of several parasites.

## METHODS TO DETECT SEX AND GENETIC EXCHANGE IN PROTISTS

Given the appearance of many protists (single celled, no obvious sexual differentiation), it has historically been difficult to detect sexual reproduction. Moreover, since vegetative growth almost always can be seen to occur, it was often assumed to be the only form of reproduction employed by protists. This was also the case for many species of fungi (Dyer and O'Gorman, 2011). However, these assumptions have, in many cases, proven to be wrong, or certainly to be not as simple as first thought.

How can sexual reproduction be inferred in a species where it is not readily apparent? There are, broadly, three ways to prove, or to infer, that a species is sexual. The clearest method, but often the most difficult, is to directly observe mating *in vitro* or *in vivo*. A second, indirect, method is to look for patterns of genetic variation in populations that indicate a sexual population structure, with out-crossing. A third indirect method is to identify genes in the genome that function in meiosis in other organisms.

### Direct identification of sex *in vivo* and *in vitro*

The clearest way to demonstrate sexual reproduction is to carry out an experimental cross to show genetic exchange. However, this can often be difficult as many parasitic protists are not easy to maintain *in vitro*. It is possible to identify morphologically distinct sexual stages in some parasites, such as *Plasmodium*, though many species do not show obvious sexual stages. It is also possible to measure the amount of nuclear DNA, which can indicate changes in ploidy as a result of meiosis. However, gametes may be difficult to recognize due to unstable ploidy in many protists, with different chromosome ploidy being present within a single nucleus (Melville *et al*. 1999; Willhoeft and Tannich, 1999; Vargas *et al.* 2004; Rogers *et al*. 2011). If sex is an obligate stage of the parasite life cycle, it may be easier to detect than if it is facultative and occurs only under specific circumstances. Among the fungi, many readily cultured species were for years thought to be exclusively asexual until recently, when sex has been demonstrated under rarely occurring conditions (O'Gorman *et al.* 2009; Dyer and O'Gorman, 2011). In some parasitic protists, it is possible that sex is facultative, which may help explain cases where the evidence is somewhat equivocal. If the species has mating incompatibility types, it may not be possible to induce mating if these are not known or included in the experiment.

### Inferring sex by identification of meiosis genes

This approach has been developed for a set of meiosis genes shown to be present in all major eukaryotic lineages (Ramesh *et al.* 2005; Malik *et al.* 2007; Schurko and Logsdon, 2008). These genes are shown in Table 1. The majority of meiosis genes have been detected in *Giardia* (Ramesh *et al.* 2005), *Trichomonas* (Malik *et al.* 2007), *Entamoeba* (Loftus *et al.* 2005; Ehrenkaufer *et al.* 2013) and *Trypanosoma, Plasmodium* and *Cryptosporidium* (Malik *et al.* 2007).

**Table 1.** Genes of the ‘meiosis detection toolkit’ and evidence of their presence in parasitic protozoal lineages. (Adapted from Malik *et al.* 2007 and Schurko and Logsdon, 2008)

Genes (paralogue)	Protein function	*Trichomonas*	*Giardia*	*Trypanosoma*	*Plasmodium*	*Cryptosporidium*	*Entamoeba*
*spo11*	Required to create DNA double-strand breaks (essential to initiate meiotic recombination among homologous chromosomes).	+	+	+	+ (2x)	+	+ (2x)
*hop1*	Synaptonemal complex protein with an important role in chromosome pairing during meiosis. Binds to double strand break regions and oligomerizes during meiotic prophase I.	+	+	+	+	+	−
*hop2* and *mnd1*	Form heterodimeric complex that interacts with RAD51 and DMC1 and promotes inter-homologue meiotic recombination and reduces synapsis and recombination of non-homologous chromosomes.	+ (2x), +	+, +	+, +	+, +	+, +	+, +
*rec8* (*rad21*)	Holds sister chromatids together during meiosis (by linking SMC1 and SMC3 subunits). Remains bound until the onset of anaphase II.	−	−	−	−	−	−
*dmc1* (*rad51*)	DMC1 and RAD51 co-localize during meiosis and cooperate during meiotic recombination. RAD51 is required for mitotic recombination, DNA damage repair, and in meiosis. DMC1 is meiosis-specific and essential for meiotic recombination and normal synaptonemal complex formation.	+	+ (2x)	+	+	+	+ (2x)
*msh4* and *msh5* (*msh2* and *msh6*)	MSH4 and MSH5 form a heterodimer and have roles in meiotic recombination and Holliday junction resolution.	+, +	−, −	+, +	−, −	−, −	+, +
*mer3*	Meiosis-specific DEAD-box helicase. Promotes Holliday junction resolution together with proteins including MSH4 and MSH5.	+	+	+	−	−	−

There are several caveats associated with using this approach to infer a species’ sexuality. Presence of the genes does not necessarily prove that a species is sexual; orthologues can have more than one function or can adapt to perform new functions in asexual species and so be maintained even in the absence of sex (reviewed in Schurko and Logsdon, 2008). The case for inferring sex from possession of meiosis genes is weakened considerably if we note that *Drosophila melanogaster* appears to lack *hop1, hop2, mnd1, dmc1, msh4, msh5* and *mer3* (see table in Malik *et al*. 2007), while species of uncertain or doubtful sexuality often possess a full or nearly full complement of meiosis genes. Conversely, it is not possible to confirm that a species is asexual based on failure to detect meiosis genes, as the orthologues could be present but highly divergent. However, while this is a problem when using PCR amplification to identify genes in the absence of a sequenced genome, an increasing number of completely sequenced genomes reduce this problem.

Additional support for the involvement of the genes in meiosis can be inferred from studying their expression pattern. In *Entamoeba histolytica* (Ehrenkaufer *et al.* 2013) and *Trypanosoma brucei* (Peacock *et al.* 2014) the expression of meiosis-specific genes coincides with developmental transitions and other evidence of meiotic events such as assortment in the case of *T. brucei* or production of tetranucleate cysts in the case of *E. histolytica*. However, in *Giardia*, up-regulation of meiosis genes is observed in the absence of meiosis (Carpenter *et al.* 2012).

### Inferring sex from population genetics

It is possible to observe the effects of genetic exchange among individuals by analysing the patterns of genetic variation within populations. True sex and mechanistically different parasexual processes of genetic exchange, as seen in bacteria, can have similar effects on patterns of population genetic variation. Clonality maintains linkage disequilibrium among polymorphic sites in the genome because there is no mechanism to break down physical linkage between alleles on the same chromosome or common descent of alleles on different chromosomes. Sex with outcrossing erodes linkage disequilibrium via recombination and re-assortment of chromosomes. However, as both asexual reproduction and sex without outcrossing will maintain linkage disequilibrium, clonal population structures do not rule out sex. The ‘clonal theory’ of parasitic protists stated that populations are primarily clonal, that is, sexual reproduction contributes very little to the population structures of these organisms (Tibayrenc *et al.* 1990; Tibayrenc and Ayala, 2002). The theory is about the relative importance of outcrossing and inbreeding and is only concerned with population structures, not cell division mechanisms (Tibayrenc and Ayala, 2013). Therefore, the failure to identify recombination may not be taken as strong evidence for the complete absence of sex. Conversely, although identification of the products of recombination is consistent with sexual reproduction in a species, it is important to remember that, as in bacteria, recombination can occur without true sex, so genetic evidence for recombination is not proof positive of the occurrence of sex.

### Sex in apicomplexan parasites

In apicomplexan parasites, sexual stages are evident and can be produced in *in vitro* culture. A sexual stage must occur to complete the life cycle. Laboratory strains of *Plasmodium falciparum* can be crossed *in vivo*, in the mosquito (Walliker *et al.* 1987). *Cryptosporidium* crosses have been described (Tanriverdi *et al.* 2007). A *Toxoplasma gondii* cross in a cat produced offspring of differing virulence (Herrmann *et al.* 2012). In *Babesia* and *Theileria* species, direct fluorimetric measurement of DNA content of parasites through the life cycle showed changes in ploidy consistent with sex in the tick gut (Mackenstedt *et al.* 1990, 1995; Gauer *et al.* 1995). Genetic exchange has been demonstrated in *Cryptosporidium parvum* (Feng *et al.* 2002).

*Plasmodium* parasites have a dimorphic sexual stage that is closely linked to the transmission cycle (the sexual stage is the transmissible stage). *Plasmodium* parasites have complex life cycles, passing through two hosts, one of which is an insect vector. The process of gametocytogenesis, in which lineages of blood stage, asexually reproducing cells switch to form male (micro-) or female (macro-) gametocytes, is intimately involved in transmission between hosts. During gametocytogenesis, the parasite changes both morphologically and biochemically (Dixon, 2008). Commitment to form gametocytes occurs one cycle before the manifestation of gametocytes (Dixon, 2008). All merozoites from a single schizont are committed to sexual/asexual pathway. Also, all merozoites from a committed schizont form *only* male or *only* female gametocytes (Silvestrini, 2000; Smith, 2000). Gametogenesis is the emergence of micro-/macro-gametocytes from infected red blood cells.

*Plasmodium falciparum* shows a range of population structures from ‘clonal’ to ‘sexual’, which broadly reflect the local transmission intensity. For instance, African populations with high transmission intensity have a greater chance of outcrossing and show very low levels of linkage disequilibrium (Conway *et al.* 1999; Anderson *et al.* 2000), while South East Asian populations with lower transmission intensity show less outcrossing and more ‘clonal’ population structures (Volkman *et al*. 2007). Population genetic analysis of the cattle parasite *Babesia bovis* from Zambia and Turkey indicate a sexual, outcrossing population structure (Simuunza *et al.* 2011). *Babesia bovis* undergoes sexual development and fertilization in the invertebrate host (Mackenstedt *et al.* 1995; Gough *et al*. 1998). Similar observations have been made in the related parasites *Theileria parva* (Gauer *et al.* 1995). It is probably unsurprising in tick-borne diseases, where a single host is likely to be bitten by multiple infected ticks, that there is a lot of scope for mixed infections where recombination will occur. The broad host-range mammalian parasite *T. gondii*, by contrast, displays a highly clonal population structure. It is possible that this reflects the fact that individuals may rarely meet in the host, unlike the situation in *Plasmodium* or enteric parasites where transmission is high and many unrelated individuals may co-exist.

In *T. gondii* the sexual cycle only occurs in cats, the definitive host. In this case the parasite will differentiate intracellularly into male microgametes and female macrogametes, which undergo fertilization to produce a diploid zygote (Ferguson *et al*. 1974, 1975; Ferguson, 2002). Unlike in *Plasmodium*, this sexual cycle has not resulted in a panmictic population structure. In fact, what is observed is three clonal lineages found in human populations while a great deal of genetic diversity can be observed in wild animals (Grigg and Sundar, 2009). Boyle *et al*. (2006) have demonstrated that the pattern of polymorphism of Types I and III *Toxoplasma* are indicative of these being first and second generation offspring of a Type II strain cross with an ancestral line. This observation highlights that a single recombination event can have a major impact on the population structure of a pathogen.

It therefore appears that in *Apicomplexa* parasites, where haploid stages are often the infective forms of the parasite, sex is common and probably ubiquitous. Clonal population structures such as those seen in *Toxoplasma* are likely caused by the rapid expansion of epidemic strains that propagate in certain hosts, in this case human.

### Sex in kinetoplastid parasites

In kinetoplastid parasites (*Trypanosoma* and *Leishmania*), sexual stages are not easily identifiable. However, genetic exchange has been identified from laboratory crosses of *T. brucei* in the tsetse fly host (MacLeod *et al.* 2005) and of *Leishmania major* in the sand fly host (Akopyants *et al.* 2009). In *T. brucei*, cell fusion has been demonstrated between cells containing red or green fluorescent proteins leading to yellow fluorescent hybrids *in vivo*, in the salivary glands of the tsetse fly host (Gibson *et al.* 2008) and *in vitro*, between cells derived from salivary glands but not between cells derived from the midgut (Peacock *et al.* 2014). Measurement of nuclear DNA content throughout the life cycle showed a haploid ‘promastigote-like’ cell in the salivary glands of the fly (Peacock *et al.* 2014) and cells expressing meiosis genes (Mnd1, Dmc1 and Hop1, see below) prior to cell fusion (Peacock *et al*. 2011, 2014). This, together with the evidence from genetic crosses, indicates that the life cycle of *T. brucei* does contain a sexual stage and meiosis.

*Trypanosoma cruzi* and *Trypanosoma vivax* appear to have all of the genes for meiosis (Malik *et al.* 2007), despite very little evidence from the population structure that meiosis is occurring (Westenberger *et al.* 2005; Duffy *et al.* 2009). Genetic exchange was demonstrated in *T. cruzi* by observing that two strains carrying different drug-selectable markers produced strains carrying both markers in the mammalian host (Gaunt *et al.* 2003). However, the proposed mechanism of this exchange was a fusion of diploid cells followed by chromosome loss, rather than meiosis; quite different to sex in *T. brucei*.

The population structure of *T. brucei* is characteristic of facultative recombination, with clonal population structures in foci of human disease and evidence of rare recombination in the wider population (Macleod *et al.* 2001). By contrast, *T. vivax* displays a clonal population structure, consistent with its more limited life cycle in the tsetse fly host where it remains in the mouthparts (Duffy *et al.* 2009). Due to its more limited life cycle *T. vivax* is able to utilize various species of biting flies as vectors which is advantageous but may have evolved at the expense of its ability to undergo meiosis and sexual recombination. In *T. cruzi*, the population structure appears to be clonal, with six major lineages circulating in human population. Recombinant strains have been described (Lewis *et al.* 2011) but whether or not these were produced by true sex is unknown. Westenberger *et al.* (2005) proposed a model whereby fusion and loss of heterozygosity between two ancestral strains could give rise to all of the major lineages observed today.

### Sex in amoebozoan parasites

*Entamoeba histolytica* is a parasitic amoeba of humans. Its life cycle has two distinct stages, a motile trophozoite that lives in the colon and a cyst that passes out of the host, can survive in the environment and be transmitted to new hosts.

*Entamoeba histolytica* was traditionally thought to be asexual, with intestinal trophozoites dividing by mitosis and encystation and excystation being mitotic too. The cysts may be formed by incomplete mitosis, as, in *E. histolytica*, they contain four nuclei (other species contain different numbers). Indirect evidence indicates sex, including possession of meiosis genes (Loftus *et al.* 2005) and population genetic evidence (Gilchrist *et al.* 2012; Weedall *et al.* 2012). A study of genomes derived from several geographical origins indicated decay in linkage with increased physical distance between variant sites (Weedall *et al.* 2012) and a set of single nucleotide polymorphism (SNP) markers in field isolates from Bangladesh showed no linkage disequilibrium at all between markers (Gilchrist *et al.* 2012), both indicative of recombination and reassortment.

If the species is sexual, when might meiosis occur? Either it must occur at some point in the transition between trophozoite or during a facultative sexual cycle under specific circumstances. During the transition from trophozoite to cyst, several meiosis genes are up-regulated (Ehrenkaufer *et al.* 2013; Singh *et al.* 2013) and homologous recombination occurs (Singh *et al.* 2013). This may indicate meiosis, as appears to be the case in *T. brucei* (Peacock *et al*. 2011, 2014), but could also indicate a non-meiotic process, as appears to be the case in *Giardia lamblia* (Carpenter *et al.* 2012).

Another (albeit distantly related) amoebazoan, *Dictyostelium discoideum*, has a sexual cycle and appears to mate frequently in wild populations (Flowers *et al*. 2010). Sex appears to be facultative and to occur under stress conditions (Saga *et al*. 1983). *Dictyostelium discoideum* has three sexes, determined by a mating type locus (Bloomfield *et al*. 2010). Haploid amoebae of different sexes fuse, attract surrounding cells and form a macrocyst in which meiosis and mitosis (presumably) occur to produce haploid cells (Saga *et al*. 1983). However, *D. discoideum* does appear to lack five meiosis genes.

### Sex in Giardia and Trichomonas parasites

Direct observation *in vitro* or *in vivo* can confirm sex, but not observing it does not provide definitive evidence that a species is asexual. For instance, in *G. lamblia* population genetic evidence has been used to infer sex (Cooper *et al*. 2007), and if sex occurs, it is thought to happen during encystation or excystation. Laboratory studies have demonstrated that nuclear division during encystation is mitotic, not meiotic (Carpenter *et al.* 2012; Jirakova *et al*. 2012). Using the distribution of different markers integrated into the genomes of two cell lines, Carpenter *et al.* (2012) were able to track the inheritance of nuclei during encystation and excystation. They found no evidence of cell fusion and also that pairs of parental nuclei were co-inherited (i.e. nuclear sorting did not occur). Several meiotic gene homologues are up-regulated during encystation, indicating that homologous recombination might occur during encystation/excystation (Carpenter *et al.* 2012). The study found evidence for ‘diplomixis’, or chromosomal genetic exchange between nuclei, and the authors argued that this, along with homologous recombination, might be sufficient to maintain the low levels of allelic heterozygosity seen in sequenced genomes (Morrison *et al*. 2007; Jerlstrom-Hultqvist *et al*. 2010). However, this model of genetic exchange cannot explain population genetic evidence for recombination within and between assemblages of *G. lamblia* (Cooper *et al.* 2007; Lasek-Nesselquist *et al*. 2009), which would require cell fusion events and some form of meiosis to occur.

The discrepancy between the evidence from *in vitro* experiments and population genetic observations of *G. lamblia* (Cooper *et al.* 2007; Carpenter *et al.* 2012) could be resolved if sex is facultative and occurs, even rarely, in natural populations. Also, if mating compatibility is determined by mating type loci, as in *D. discoideum*, the *in vitro* experiments may not show any mating if a single strain is used.

*Trichomonas vaginalis* is a parabasalid parasite of humans that is sexually transmitted. Two major lineages, both with a global distribution, can be defined by microsatellite patterns, yet within populations there is little genome wide linkage disequilibrium, suggesting that recombination is breaking linkage (Conrad *et al.* 2012).

## SEXUAL STAGES AND TRANSMISSION BETWEEN HOSTS

In many different species, meiosis and recombination are linked to dispersal. Either gametes or dormant zygotes (or seeds) are dispersed to find new environmental niches. Dispersal (transmission) is central to the parasitic lifestyle. Cysts, often the transmissible stages in parasitic species, may have occurred in early eukaryotes and may be linked to the evolution of sex (Cavalier-Smith, 2002). However, cysts and cyst-like stages may be examples of convergent evolution, as the materials forming cyst walls differ among species and the encystation process may also be very different (Lauwaet *et al.* 2007; Ehrenkaufer *et al.* 2013; Samuelson *et al.* 2013). We may identify a link between sex and transmission in parasites.

To illustrate how the sexual stages of parasites can be linked to their life cycle and adapt to their differing lifestyles, consider two greatly different modes of transmission in parasites of the *Apicomplexa*: transmission via a vector species and direct transmission via the fecal–oral route. Some apicomplexans, including *Plasmodium* and *Babesia* species, are transmitted from a vertebrate host to an insect or tick host and to another vertebrate host. Others, including *Cryptosporidium, Eimeria* and *Toxoplasma* species, are transmitted among hosts via an environmental stage (the oocyst). The requirements of these modes of transmission are different and the sexual stages appear to be adapted accordingly. In the environmentally transmitted parasites (e.g. *Eimeria*), oocysts are tough structures allowing survival outside of a host. Gametocytogenesis and gametogenesis form a continuous process that takes place inside one infected host cell, and appears to be programmed to occur after approximately three asexual cycles. In contrast, vector borne parasites must negotiate a novel host. *Plasmodium* and *Babesia* parasites display a pause between gametocytogenesis and gametogenesis so that gametogenesis occurs after transmission, within the gut of the mosquito/tick host, rather than in the vertebrate host. *Plasmodium* parasites have a stage not seen in fecal–oral transmitted parasites: the ookinete is formed after gamete fusion, it crosses the insect gut wall before forming the oocyst. In contrast to the apparently timed triggering of sexual commitment in *Eimeria*, in *Plasmodium*, commitment to sexual development appears to be triggered by environment and to be ‘staggered’ to facilitate transmission. Producing transmissible stages over a longer time makes sense for vector borne parasites to increase the chances of encountering a vector. In fecal–oral transmitted parasites, transmissible stages will be passed into the environment in any case and controlling of the timing of their production is likely to be less important than their ability to survive once there.

### Concluding remarks

Understanding the sexual biology of parasitic protists is not only of intrinsic scientific interest but may also be biomedically relevant, informing potential treatments to target sexual stages of the parasites. For instance, knowing that sex is necessary for apicomplexan parasites to complete their life cycles makes sexual stages potential targets for intervention. By contrast, facultative sexual stages may not be useful targets. Biological knowledge of the sexual stages, in particular the comparative biology of sex, may allow for the identification of conserved target processes, molecules or structures (e.g. proteins mediating cell binding or flagella in microgametes (Wallach *et al.* 1995, 2008). Therefore, improving our understanding of sex in parasitic protists is an important goal for future parasitological study.

## References

[ref1] Akopyants N. S., Kimblin N., Secundino N., Patrick R., Peters N., Lawyer P., Dobson D. E., Beverley S. M. and Sacks D. L. (2009). Demonstration of genetic exchange during cyclical development of *Leishmania* in the sand fly vector. Science 324, 265–268. 19359589 10.1126/science.1169464 PMC2729066

[ref2] Anderson T. J., Haubold B., Williams J. T., Estrada-Franco J. G., Richardson L., Mollinedo R., Bockarie M., Mokili J., Mharakurwa S., French N., Whitworth J., Velez I. D., Brockman A. H., Nosten F., Ferreira M. U. and Day K. P. (2000). Microsatellite markers reveal a spectrum of population structures in the malaria parasite *Plasmodium falciparum*. Molecular and Biochemical Evolution 17, 1467–1482. 10.1093/oxfordjournals.molbev.a026247 11018154

[ref3] Baldauf S. L. (2003). The deep roots of eukaryotes. Science 300, 1703–1706. 12805537 10.1126/science.1085544

[ref4] Bloomfield G., Skelton J., Ivens A., Tanaka Y. and Kay R. R. (2010). Sex determination in the social amoeba *Dictyostelium discoideum*. Science 330, 1533–1536. 21148389 10.1126/science.1197423 PMC3648785

[ref5] Boyle J. P., Rajasekar B., Saeij J. P., Ajioka J. W., Berriman M., Paulsen I., Roos D. S., Sibley L. D., White M. W. and Boothroyd J. C. (2006). Just one cross appears capable of dramatically altering the population biology of a eukaryotic pathogen like *Toxoplasma gondii*. Proceedings of the National Academy of Sciences USA 103, 10514–10519. 10.1073/pnas.0510319103 PMC1502489 16801557

[ref6] Carpenter M. L., Assaf Z. J., Gourguechon S. and Cande W. Z. (2012). Nuclear inheritance and genetic exchange without meiosis in the binucleate parasite Giardia intestinalis. Journal of Cell Science 125, 2523–2532. 22366460 10.1242/jcs.103879 PMC3383261

[ref7] Cavalier-Smith T. (2002). Origins of the machinery of recombination and sex. Heredity 88, 125–141. 11932771 10.1038/sj.hdy.6800034

[ref8] Cavalier-Smith T. (2010). Origin of the cell nucleus, mitosis and sex: roles of intracellular coevolution. Biology Direct 5, 7. 20132544 10.1186/1745-6150-5-7 PMC2837639

[ref69] Cooper M. A., Adam R. D., Worobey M. and Sterling C. R. (2007). Population genetics provides evidence for recombination in Giardia. Curr Biol. Nov 20;17(22):1984–8. Epub 2007 Nov 1. PubMed PMID: 17980591. 17980591 10.1016/j.cub.2007.10.020

[ref9] Conrad M. D., Gorman A. W., Schillinger J. A., Fiori P. L., Arroyo R., Malla N., Dubey M. L., Gonzalez J., Blank S., Secor W. E. and Carlton J. M. (2012). Extensive genetic diversity, unique population structure and evidence of genetic exchange in the sexually transmitted parasite *Trichomonas vaginalis*. PLoS Neglected Tropical Diseases 6, . 10.1371/journal.pntd.0001573 PMC3313929 22479659

[ref10] Conway D. J., Roper C., Oduola A. M., Arnot D. E., Kremsner P. G., Grobusch M. P., Curtis C. F. and Greenwood B. M. (1999). High recombination rate in natural populations of *Plasmodium falciparum*. Proceedings of the National Academy of Science, USA 96, 4506–4511. 10.1073/pnas.96.8.4506 PMC16362 10200292

[ref11] Cooper M. A., Adam R. D., Worobey M. and Sterling C. R. (2007). Population genetics provides evidence for recombination in *Giardia*. Current Biology 17, 1984–1988. 17980591 10.1016/j.cub.2007.10.020

[ref12] Delmotte F., Leterme N., Bonhomme J., Rispe C. and Simon J. C. (2001). Multiple routes to asexuality in an aphid species. Proceedings of the Royal Society, London B 268, 2291–2299. 10.1098/rspb.2001.1778 PMC1088879 11703868

[ref60] Dixon M. W., Thompson J., Gardiner D. L. and Trenholme K. R. (2008). Sex in Plasmodium: a sign of commitment. Trends Parasitol. Apr;24(4):168–75. doi:10.1016/j.pt.2008.01.004. Epub 2008 Mar 14. Review. PubMed PMID: 18342574. 18342574

[ref13] Duffy C. W., Morrison L. J., Black A., Pinchbeck G. L., Christley R. M., Schoenefeld A., Tait A., Turner C. M. and MacLeod A. (2009). *Trypanosoma vivax* displays a clonal population structure. International Journal of Parasitology 39, 1475–1483. 19520081 10.1016/j.ijpara.2009.05.012

[ref14] Dyer P. S. and O'Gorman C. M. (2011). A fungal sexual revolution: *Aspergillus* and *Penicillium* show the way. Current Opinion in Microbiology 14, 649–654. 22032932 10.1016/j.mib.2011.10.001

[ref15] Ehrenkaufer G. M., Weedall G. D., Williams D., Lorenzi H. A., Caler E., Hall N. and Singh U. (2013). The genome and transcriptome of the enteric parasite *Entamoeba invadens*, a model for encystation. Genome Biology 14, R77. 23889909 10.1186/gb-2013-14-7-r77 PMC4053983

[ref16] Feng X., Rich S. M., Tzipori S. and Widmer G. (2002). Experimental evidence for genetic recombination in the opportunistic pathogen *Cryptosporidium parvum*. Molecular and Biochemical Parasitology 119, 55–62. 11755186 10.1016/s0166-6851(01)00393-0

[ref65] Ferguson D. J., Hutchison W. M., Dunachie J. F. and Siim J. C. (1974). Ultrastructural study of early stages of asexual multiplication and microgametogony of Toxoplasma gondii in the small intestine of the cat. Acta Pathol Microbiol Scand B Microbiol Immunol. Apr;82(2):167–81. PubMed PMID: 4528099. 4528099 10.1111/j.1699-0463.1974.tb02309.x

[ref66] Ferguson D. J., Hutchison W. M. and Siim J. C. (1975). The ultrastructural development of the macrogamete and formation of the oocyst wall of Toxoplasma gondii. Acta Pathol Microbiol Scand B. Oct;83(5):491–505. PubMed PMID: 1180061. 1180061 10.1111/j.1699-0463.1975.tb00130.x

[ref67] Ferguson D. J. (2002). Toxoplasma gondii and sex: essential or optional extra? Trends Parasitol. Aug;18(8):355–9. PubMed PMID: 12380023. 12380023

[ref17] Flowers J. M., Li S. I., Stathos A., Saxer G., Ostrowski E. A., Queller D. C., Strassmann J. E. and Purugganan M. D. (2010). Variation, sex, and social cooperation: molecular population genetics of the social amoeba *Dictyostelium discoideum*. PLoS Genetics 6, . 10.1371/journal.pgen.1001013 PMC2895654 20617172

[ref19] Gauer M., Mackenstedt U., Mehlhorn H., Schein E., Zapf F., Njenga E., Young A. and Morzaria S. (1995). DNA measurements and ploidy determination of developmental stages in the life cycles of *Theileria annulata* and *T. parva*. Parasitology Research 81, 565–574. 7479648 10.1007/BF00932023

[ref20] Gaunt M. W., Yeo M., Frame I. A., Stothard J. R., Carrasco H. J., Taylor M. C., Mena S. S., Veazey P., Miles G. A., Acosta N., de Arias A. R. and Miles M. A. (2003). Mechanism of genetic exchange in American trypanosomes. Nature 421, 936–939. 12606999 10.1038/nature01438

[ref21] Gibson W., Peacock L., Ferris V., Williams K. and Bailey M. (2008). The use of yellow fluorescent hybrids to indicate mating in *Trypanosoma brucei*. Parasites and Vectors 1, 4. 18298832 10.1186/1756-3305-1-4 PMC2276490

[ref22] Gilchrist C. A., Ali I. K., Kabir M., Alam F., Scherbakova S., Ferlanti E., Weedall G. D., Hall N., Haque R., Petri W. A. Jr. and Caler E. (2012). A multilocus sequence typing system (MLST) reveals a high level of diversity and a genetic component to *Entamoeba histolytica* virulence. BMC Microbiology 12, 151. 22839995 10.1186/1471-2180-12-151 PMC3438053

[ref64] Gough J. M., Jorgensen W. K. and Kemp D. H. (1998). Development of tick gut forms of Babesia bigemina in vitro. J Eukaryot Microbiol. May–Jun;45(3):298–306. PubMed PMID: 9669864. 9669864 10.1111/j.1550-7408.1998.tb04540.x

[ref23] Grigg M. E. and Sundar N. (2009). Sexual recombination punctuated by outbreaks and clonal expansions predicts *Toxoplasma gondii* population genetics. International Journal of Parasitology 39, 925–933. 19217909 10.1016/j.ijpara.2009.02.005 PMC2713429

[ref24] Herrmann D. C., Bärwald A., Maksimov A., Pantchev N., Vrhovec M. G., Conraths F. J. and Schares G. (2012). Toxoplasma gondii sexual cross in a single naturally infected feline host: generation of highly mouse-virulent and avirulent clones, genotypically different from clonal types I, II and III. Veterinary Research 43, 39. 22546040 10.1186/1297-9716-43-39 PMC3443434

[ref25] Innes D. J. and Hebert P. D. N. (1988). The origin and genetic basis of obligate parthenogenesis in *Daphnia pulex*. Evolution 42, 1024–1035. 10.1111/j.1558-5646.1988.tb02521.x 28581165

[ref72] Jerlström-Hultqvist J., Franzén O., Ankarklev J., Xu F., Nohýnková E., Andersson J. O., Svärd S. G. and Andersson B. (2010). Genome analysis and comparative genomics of a Giardia intestinalis assemblage E isolate. BMC Genomics. Oct 7;11:543. doi:10.1186/1471-2164-11-543. PubMed PMID: 20929575; PubMed Central PMCID: PMC3091692. PMC3091692 20929575

[ref70] Jiráková K., Kulda J. and Nohýnková E. (2012). How nuclei of Giardia pass through cell differentiation: semi-open mitosis followed by nuclear interconnection. Protist. May;163(3):465–79. doi:10.1016/j.protis.2011.11.008. Epub 2011 Dec 30. PubMed PMID: 22209008. 22209008

[ref73] Lasek-Nesselquist E., Welch D. M., Thompson R. C., Steuart R. F. and Sogin M. L. (2009). Genetic exchange within and between assemblages of Giardia duodenalis. J Eukaryot Microbiol. Nov–Dec;56(6):504–18. doi: 10.1111/j.1550-7408.2009.00443.x Erratum in: *J Eukaryot Microbiol.* 2010 Jan 1;57(1):94. PubMed PMID: 19883439. 19883439

[ref27] Lauwaet T., Davids B. J., Reiner D. S. and Gillin F. D. (2007). Encystation of *Giardia lamblia*: a model for other parasites. Current Opinion in Microbiology 10, 554–559. 17981075 10.1016/j.mib.2007.09.011 PMC2709507

[ref28] Lehtonen J., Jennions M. D. and Kokko H. (2012). The many costs of sex. Trends in Ecology and Evolution 27, 172–178. 22019414 10.1016/j.tree.2011.09.016

[ref29] Lewis M. D., Llewellyn M. S., Yeo M., Acosta N., Gaunt M. W. and Miles M. A. (2011). Recent, independent and anthropogenic origins of *Trypanosoma cruzi* hybrids. PLoS Neglected Tropical Diseases 5, . 10.1371/journal.pntd.0001363 PMC3191134 22022633

[ref30] Loftus B., Anderson I., Davies R., Alsmark U. C., Samuelson J., Amedeo P., Roncaglia P., Berriman M., Hirt R. P., Mann B. J., Nozaki T., Suh B., Pop M., Duchene M., Ackers J., Tannich E., Leippe M., Hofer M., Bruchhaus I., Willhoeft U., Bhattacharya A., Chillingworth T., Churcher C., Hance Z., Harris B., Harris D., Jagels K., Moule S., Mungall K., Ormond D. (2005). The genome of the protist parasite *Entamoeba histolytica*. Nature 433, 865–868. 15729342 10.1038/nature03291

[ref31] Mackenstedt U., Gauer M., Mehlhorn H., Schein E. and Hauschild S. (1990). Sexual cycle of *Babesia divergens* confirmed by DNA measurements. Parasitology Research 76, 199–206. 2315280 10.1007/BF00930815

[ref32] Mackenstedt U., Gauer M., Fuchs P., Zapf F., Schein E. and Mehlhorn H. (1995). DNA measurements reveal differences in the life cycles of *Babesia bigemina* and *B. canis*, two typical members of the genus *Babesia*. Parasitology Research 81, 595–604. 7479652 10.1007/BF00932027

[ref33] MacLeod A., Tait A. and Turner C. M. (2001). The population genetics of *Trypanosoma brucei* and the origin of human infectivity. Philosophical Transactions of the Royal Society of London, B (Biological Science) 356, 1035–1344. 10.1098/rstb.2001.0892 PMC1088498 11516381

[ref34] MacLeod A., Tweedie A., McLellan S., Hope M., Taylor S., Cooper A., Sweeney L., Turner C. M. and Tait A. (2005). Allelic segregation and independent assortment in *T. brucei* crosses: proof that the genetic system is Mendelian and involves meiosis. Molecular Biochemical Parasitology 143, 12–19. 15941603 10.1016/j.molbiopara.2005.04.009

[ref35] Malik S. B., Pightling A. W., Stefaniak L. M., Schurko A. M. and Logsdon J. M. Jr. (2007). An expanded inventory of conserved meiotic genes provides evidence for sex in *Trichomonas vaginalis*. PLoS ONE 3, . 10.1371/journal.pone.0002879 PMC2488364 18663385

[ref36] Melville S. E., Gerrard C. S. and Blackwell J. M. (1999). Multiple causes of size variation in the diploid megabase chromosomes of African trypanosomes. Chromosome Research 7, 191–203. 10421379 10.1023/a:1009247315947

[ref71] Morrison H. G., McArthur A. G., Gillin F. D., Aley S. B., Adam R. D., Olsen G. J., Best A. A., Cande W. Z., Chen F., Cipriano M. J., Davids B. J., Dawson S. C., Elmendorf H. G., Hehl A. B., Holder M. E., Huse S. M., Kim U. U., Lasek-Nesselquist E., Manning G., Nigam A., Nixon J. E., Palm D., Passamaneck N. E., Prabhu A., Reich C. I., Reiner D. S., Samuelson J., Svard S. G. and Sogin M. L. (2007). Genomic minimalism in the early diverging intestinal parasite Giardia lamblia. Science. Sep 28;317(5846):1921–6. PubMed PMID: 17901334. 17901334 10.1126/science.1143837

[ref38] O'Gorman C. M., Fuller H. and Dyer P. S. (2009). Discovery of a sexual cycle in the opportunistic fungal pathogen *Aspergillus fumigatus*. Nature 457, 471–474. 19043401 10.1038/nature07528

[ref39] Peacock L., Bailey M., Carrington M. and Gibson W. (2014). Meiosis and haploid gametes in the pathogen *Trypanosoma brucei*. Current Biology 24, 181–186. 24388851 10.1016/j.cub.2013.11.044 PMC3928991

[ref68] Peacock L., Ferris V., Sharma R., Sunter J., Bailey M., Carrington M. and Gibson W. (2011). Identification of the meiotic life cycle stage of Trypanosoma brucei in the tsetse fly. Proc Natl Acad Sci USA. Mar 1;108(9):3671–6. doi:10.1073/pnas.1019423108. Epub 2011 Feb 14. PubMed PMID: 21321215; PubMed Central PMCID: PMC3048101. 21321215 PMC3048101

[ref40] Ramesh M. A., Malik S. B. and Logsdon J. M. Jr. (2005). A phylogenomic inventory of meiotic genes; evidence for sex in *Giardia* and an early eukaryotic origin of meiosis. Current Biology 15, 185–191. 15668177 10.1016/j.cub.2005.01.003

[ref41] Rogers M. B., Hilley J. D., Dickens N. J., Wilkes J., Bates P. A., Depledge D. P., Harris D., Her Y., Herzyk P., Imamura H., Otto T. D., Sanders M., Seeger K., Dujardin J. C., Berriman M., Smith D. F., Hertz-Fowler C. and Mottram J. C. (2011). Chromosome and gene copy number variation allow major structural change between species and strains of *Leishmania*. Genome Research 21, 2129–2142. 22038252 10.1101/gr.122945.111 PMC3227102

[ref42] Saga Y., Okada H. and Yanagisawa K. (1983). Macrocyst development in *Dictyostelium discoideum*. II. Mating-type specific cell fusion and acquisition of fusion-competence. Journal of Cell Science 60, 157. 6874727 10.1242/jcs.60.1.157

[ref43] Samuelson J., Bushkin G. G., Chatterjee A. and Robbins P. W. (2013). Strategies to discover the structural components of cyst and oocyst walls. Eukaryotic Cell 12, 1578–1587. 24096907 10.1128/EC.00213-13 PMC3889564

[ref44] Schurko A. M. and Logsdon J. M. (2008). Using a meiosis detection toolkit to investigate ancient asexual “scandals” and the evolution of sex. Bioessays 30, 579–589. 18478537 10.1002/bies.20764

[ref61] Silvestrini F., Alano P. and Williams J. L. (2000). Commitment to the production of male and female gametocytes in the human malaria parasite Plasmodium falciparum. Parasitology. Nov;121 Pt 5:465–71. PubMed PMID: 11128797. 11128797 10.1017/s0031182099006691

[ref45] Simuunza M., Bilgic H., Karagenc T., Syakalima M., Shiels B., Tait A. and Weir W. (2011). Population genetic analysis and sub-structuring in *Babesia bovis*. Molecular and Biochemical Parasitology 177, 106–115. 21316400 10.1016/j.molbiopara.2011.02.002

[ref46] Singh N., Bhattacharya A. and Bhattacharya S. (2013). Homologous recombination occurs in *Entamoeba* and is enhanced during growth stress and stage conversion. PLoS ONE 8, . 10.1371/journal.pone.0074465 PMC3787063 24098652

[ref63] Smith T. G., Lourenço P., Carter R., Walliker D. and Ranford-Cartwright L. C. (2000). Commitment to sexual differentiation in the human malaria parasite, Plasmodium falciparum. Parasitology. Aug;121 ( Pt 2):127–33. PubMed PMID: 11085232. 11085232 10.1017/s0031182099006265

[ref47] Tanriverdi S., Blain J. C., Deng B., Ferdig M. T. and Widmer G. (2007). Genetic crosses in the apicomplexan parasite *Cryptosporidium parvum* define recombination parameters. Molecular Microbiology 63, 1432–1439. 17302818 10.1111/j.1365-2958.2007.05594.x

[ref48] Tibayrenc M. and Ayala F. J. (2002). The clonal theory of parasitic protozoa: 12 years on. Trends in Parasitology 18, 405–410. 12377258 10.1016/s1471-4922(02)02357-7

[ref49] Tibayrenc M. and Ayala F. J. (2013). How clonal are *Trypanosoma* and *Leishmania*? Trends in Parasitology 29, 264–269. 23602631 10.1016/j.pt.2013.03.007

[ref50] Tibayrenc M. and Ayala F. J. (2014). New insights into clonality and panmixia in *Plasmodium* and toxoplasma. Advances in Parasitology 84, 253–268. 24480316 10.1016/B978-0-12-800099-1.00005-3

[ref51] Tibayrenc M., Kjellberg F. and Ayala F. J. (1990). A clonal theory of parasitic protozoa: the population structures of *Entamoeba, Giardia, Leishmania, Naegleria, Plasmodium, Trichomonas*, and *Trypanosoma* and their medical and taxonomical consequences. Proceedings of the National Academy of Science USA 87, 2414–2418. 10.1073/pnas.87.7.2414 PMC53699 2320563

[ref52] Wallach M., Smith N. C., Petracca M., Miller C. M., Eckert J. and Braun R. (1995). *Eimeria maxima* gametocyte antigens: potential use in a subunit maternal vaccine against coccidiosis in chickens. Vaccine 13, 347–354. 7793129 10.1016/0264-410x(95)98255-9

[ref53] Wallach M. G., Ashash U., Michael A. and Smith N. C. (2008). Field application of a subunit vaccine against an enteric protozoan disease. PLoS ONE 3, . 10.1371/journal.pone.0003948 PMC2596963 19079606

[ref54] Vargas N., Pedroso A. and Zingales B. (2004). Chromosomal polymorphism, gene synteny and genome size in *T. cruzi* I and *T. cruzi* II groups. Molecular and Biochemical Parasitology 138, 131–141. 15500924 10.1016/j.molbiopara.2004.08.005

[ref55] Volkman S. K., Sabeti P. C., DeCaprio D., Neafsey D. E., Schaffner S. F., Milner D. A. Jr., Daily J. P., Sarr O., Ndiaye D., Ndir O., Mboup S., Duraisingh M. T., Lukens A., Derr A., Stange-Thomann N., Waggoner S., Onofrio R., Ziaugra L., Mauceli E., Gnerre S., Jaffe D. B., Zainoun J., Wiegand R. C., Birren B. W., Hartl D. L., Galagan J. E., Lander E. S. and Wirth D. F. (2007). A genome-wide map of diversity in *Plasmodium falciparum*. Nature Genetics 39, 113–119. 17159979 10.1038/ng1930

[ref56] Walliker D., Quakyi I. A., Wellems T. E., McCutchan T. F., Szarfman A., London W. T., Corcoran L. M., Burkot T. R. and Carter R. (1987). Genetic analysis of the human malaria parasite *Plasmodium falciparum*. Science 236, 1661–1666. 3299700 10.1126/science.3299700

[ref57] Weedall G. D., Clark C. G., Koldkjaer P., Kay S., Bruchhaus I., Tannich E., Paterson S. and Hall N. (2012). Genomic diversity of the human intestinal parasite *Entamoeba histolytica*. Genome Biology 13, R38. 22630046 10.1186/gb-2012-13-5-r38 PMC3446291

[ref58] Westenberger S. J., Barnabé C., Campbell D. A. and Sturm N. R. (2005). Two hybridization events define the population structure of *Trypanosoma cruzi*. Genetics 171, 527–543. 15998728 10.1534/genetics.104.038745 PMC1456769

[ref59] Willhoeft U. and Tannich E. (1999). The electrophoretic karyotype of *Entamoeba histolytica*. Molecular and Biochemical Parasitology 99, 41–53. 10215023 10.1016/s0166-6851(98)00178-9

